# Hemodynamic Modeling and Phase‐Adjustable Reconstruction of Hyperpolarized Cardiac ^13^C MRS Using ^1^H Cine and ECG Timing

**DOI:** 10.1002/mrm.70426

**Published:** 2026-05-10

**Authors:** Sung‐Han Lin, Corey Mozingo, Crystal E. Harrison, Kelley A. Derner, Craig R. Malloy, Jae Mo Park

**Affiliations:** ^1^ Advanced Imaging Research Center UT Southwestern Medical Center Dallas Texas USA; ^2^ VA North Texas Healthcare System Dallas Texas USA; ^3^ Department of Radiology UT Southwestern Medical Center Dallas Texas USA; ^4^ Department of Biomedical Engineering UT Southwestern Medical Center Dallas Texas USA; ^5^ Charles and Jane Pak Center for Mineral Metabolism and Clinical Research UT Southwestern Medical Center Dallas Texas USA

**Keywords:** cardiac metabolism, hemodyanmics, human heart, hyperpolarized pyruvate, kinetic modeling

## Abstract

**Purpose:**

To deconvolve cardiac phase and hemodynamic effects in hyperpolarized (HP) [1‐^13^C]pyruvate and to develop a cardiac phase‐adjustable reconstruction framework.

**Methods:**

Cardiac phase drift during dynamic acquisition of HP ^13^C MRS and its effect on dynamic signal fidelity were simulated using a digital cardiac phantom. A hemodynamic model describing ventricular passages of HP pyruvate was developed and applied to dynamic cardiac ^13^C MRS datasets acquired from healthy volunteers. Timecourses of HP pyruvate from multiphase ^13^C MRI and ^13^C MRS were fit to the hemodynamic model and were compared with multiphase ^1^H and ECG timing. Phase‐adjustable reconstruction was demonstrated for ECG‐gated and nongated HP ^13^C MRS.

**Results:**

Phantom simulations demonstrated that heart rate variability induces cardiac phase misalignment, causing substantial distortion of dynamic HP pyruvate timecourses. Human studies showed transient heart rate changes during pyruvate bolus arrival, resulting in phase shifts. Multiphase ^13^C MRI confirmed that pyruvate signal is correlated with ventricular volume (*R* ^*2*^ = 0.88 ± 0.08), with diastolic signals exceeding systolic signals in both ventricles and LV peak less than half of RV. The hemodynamic model accurately depicted in vivo cardiac ^13^C MRS (*R* ^*2*^ > 0.978), separating LV, RV, and recirculation components and estimating pulmonary transit times consistent with image‐based measurements. Retrospective phase correction enabled reconstruction of dynamic HP pyruvate timecourses aligned to specific cardiac phases.

**Conclusion:**

We present a volume‐guided hemodynamic modeling and reconstruction framework that corrects cardiac phase misalignment. This approach improves quantitative robustness across varying heart rates and is applicable to both ECG‐gated and nongated acquisitions.

## 1 Introduction

Pyruvate sits at a pivotal node in mammalian metabolism and plays a central role in metabolic reprogramming under a wide range of pathophysiological conditions. Carbon‐13 (^13^C) MRI with bolus‐injected hyperpolarized (HP) ^13^C‐labeled pyruvate has become a powerful modality for capturing real‐time pyruvate metabolism in vivo [1]. The substantial, yet transient ^13^C signal enhancement achieved through hyperpolarization is preserved as pyruvate is enzymatically converted to downstream metabolites. Singly ^13^C‐labeled pyruvate in the carboxyl group ([1‐^13^C]pyruvate) has been widely utilized because its long *T*_*1*_ relaxation time allows for an extended observation window for detecting its products such as [1‐^13^C]lactate, [1‐^13^C]alanine, and [^13^C]bicarbonate. Its utility for detecting aberrant metabolism in cancers, cardiovascular diseases, neurological disorders, and metabolic syndromes has been showcased in both preclinical studies and clinical investigations.

In cardiac applications, HP pyruvate ^13^C MRI/MRS offers metabolic insights [2, 3] that complement standard ^1^H cardiac MRI [4], which assesses cardiac structure and function through multiphase (cine) imaging, myocardial perfusion imaging, and late‐gadolinium enhancement (LGE) imaging. In particular, the appearance of HP [^13^C]bicarbonate from HP [1‐^13^C]pyruvate has been associated with altered metabolic flux through pyruvate dehydrogenase (PDH) in multiple cardiomyopathies such as myocardial infarction [2, 5], diabetic cardiomyopathy [6, 7], heart failure [8, 9], and cardiotoxicity [10, 11]. These findings support the clinical promise of HP pyruvate as a tool for assessing in vivo myocardial metabolism.

Despite these advances, quantitative metrics for cardiac HP imaging remain poorly defined. Metrics used in noncardiac applications, including kinetic parameters such as apparent conversion rate from pyruvate to lactate (*k*_*PL*_), normalized product area under the curve (AUC) to pyruvate AUC or total HP signals AUC, or product‐to‐product ratios, are not directly applicable to the heart for several cardiac‐specific challenges. First, cyclic cardiac motion introduces substantial variability in cardiac HP ^13^C data. Distinct myocardial metabolic profiles measured using HP ^13^C pyruvate have been reported between end‐systole and end‐diastole [12]. Although electrocardiogram (ECG) gating mitigates motion‐related artifacts, heart rate (HR) variability is often exacerbated by pyruvate bolus injection, leading to cardiac‐phase misalignments across timepoints. Second, HP signal components arise from distinct anatomical compartments; pyruvate is detected predominantly within the ventricular cavity, bicarbonate arises from the myocardium, and lactate may originate from both. Third, the temporal resolution of typical HP cardiac protocols is insufficient to fully capture the rapid first‐pass kinetics of intravenously administered pyruvate, a limitation that becomes even more pronounced in metabolite‐interleaved or multiecho acquisition schemes.

To enable robust quantification of HP cardiac metabolism, an improved understanding of the underlying HP injectate dynamics is required. Such characterization must incorporate the interplay among *T*_*1*_ decay, the arterial input function (AIF), and cardiac hemodynamics. Accordingly, there is a critical need for a comprehensive model that delineates the unique kinetic and physiological features of HP pyruvate in the heart. In this study, we develop a hemodynamic model for cardiac HP pyruvate dynamics in vivo, accounting for compartment‐specific contributions and cardiac‐phase dependence. Building on this model, we introduce a reconstruction framework that corrects cardiac‐phase misalignment and enables estimation of HP pyruvate signals at consistent or user‐defined cardiac phases.

## 2 Methods

### 2.1 Hemodynamic Modeling of HP [1‐ ^13^C]Pyruvate in the Heart Using a Digital Phantom

The impact of cardiac phase and HR variability on the dynamic HP signal was simulated using a numerical cardiac phantom. The digital phantom was adapted from the MRXCAT framework [13]. A 20‐phase cardiac phantom was created for a single slice along the short‐axis plane (matrix size = 204 × 307, voxel size = 1 × 1 mm^2^, TR/TE = 2000/1.5 ms, SNR = 80 dB). The left ventricle (LV), right ventricle (RV), and myocardium were segmented for each frame to derive dynamic volume changes of the compartments, and LV and RV volume curves were generated using periodic spline interpolation. This single‐slice approach was chosen to provide a computationally efficient, representative profile of fractional ventricular volume changes sufficient for demonstrating phase‐shift artifacts.

The AIF in each ventricle was described by a gamma‐variate function, accounting for bolus dispersion, multiplied by a mono‐exponential decay representing in vivo *T*_*1*_ relaxation of HP signals. The first‐pass AIF, *f(t)*, for each ventricle was defined as Equation (1), where $t_{a}$ is the arrival time of pyruvate bolus at the region of interest, *k* is the dimensionless shape parameter, θ is the scale parameter, and T is in vivo *T*_*1*_ of [1‐^13^C]pyruvate, previously estimated to be 29 s [14]. The dynamic timecourse signal of HP [1‐^13^C]pyruvate within the heart was modeled as a superposition of signals from the blood pools in both ventricles. $t_{a}$ was estimated as 10 s for the RV curve to reflect a 15‐s bolus injection [15, 16], and an additional delay of 6.7 s was applied to the LV curve to account for pulmonary transit time (PTT) at an average HR of 59 bpm [17].

(1) $$f\left(t\right)=\frac{{\left(t-t_{a}\right)}^{k-1}}{\Gamma \left(k\right)\cdot \theta^{k}}\cdot e^{-\left(t-t_{a}\right)\cdot \left(\frac{1}{\theta }+\frac{1}{T}\right)},t\geq t_{a}$$

### 2.2 Simulation of Cardiac Dynamic Signal Curves With Phase Misalignment

The effects of HR variation and the resulting cardiac phase misalignment on the dynamic HP [1‐^13^C]pyruvate signal were simulated by comparing *misaligned* timecourses obtained from nongated or mis‐triggered acquisition with *phase‐aligned* timecourses at end‐systole. Phase‐aligned dynamic curves were first generated for a range of HRs from 38 to 101 bpm in 7‐bpm increments. The PTT was modeled as inversely proportional to the HR, and the PTT for a given HR was calculated using Equation (2). The reference HR (${\mathrm{HR}}_{\mathrm{ref}}$) was set to 59 bpm for 6.7 s of PTT.

(2) $$\begin{array}{c} \mathrm{PTT}\left(\mathrm{HR}\right)={\mathrm{PTT}}_{\mathrm{ref}}\times \frac{{\mathrm{HR}}_{\mathrm{ref}}}{\mathrm{HR}} \end{array}$$

From these phase‐aligned timecourses, misaligned timecourses were synthesized using a sampling period of 2 s, mimicking a non‐ECG‐gated acquisition. For each HR, progressive cardiac phase shifts were calculated over time. Signal intensity was then modulated by a factor derived from the ratio of ventricular volume at the actual (drifted) cardiac phase to that at the ideal end‐systolic (ES) phase.

### 2.3 Study Participants and MR Protocols

The human study protocol was approved by the local Institutional Review Board (IRB#: STU 012011‐070). Two healthy volunteers were recruited, and written informed consent was obtained. The study was performed in accordance with the Declaration of Helsinki. Participant #1 (26 years old, Asian male) received three separate HP [1‐^13^C]pyruvate injections for two nongated dynamic ^13^C MRS and a five‐phase ECG‐gated ^13^C MRI of pyruvate. Participant #2 (30 years old, White female) received two HP pyruvate injections for a nongated and a prospectively ECG‐gated dynamic ^13^C MRS. Participant information and study protocol are summarized in Table 1.

**TABLE 1:** Subject information and experimental design.

					^1^H cine MRI	HP ^13^C MRS/MRI		
ID	Gender	Age	Ethnicity	BMI	#phases	Injection#1	Injection#2	Injection#3
1	Male	26	Asian	23.6	11	MRS, nongated	MRS, nongated	Multiphase pyruvate MRI, ECG‐gated
2	Female	30	White	30.0	13	MRS, nongated	MRS, ECG‐gated	—

All MR studies were performed on a clinical 3 T wide bore MRI scanner (750 W Discovery; GE Healthcare, Waukesha, WI, USA). The body coil was used for ^1^H imaging, and a 20‐cm diameter two‐loop ^13^C transmit/receive coil (PulseTeq Limited, Chobham, Surrey, UK) was used for ^13^C acquisition. Cardiac gating was performed using an ECG, and the timing of each RF excitation was recorded. A 5‐T dynamic nuclear polarization (DNP) clinical polarizer (SPINlab, GE Healthcare) was used to prepare HP pyruvate samples, as described previously [18]. Each subject received consecutive bolus injections of a 250‐mM HP [1‐^13^C]pyruvate solution (0.4 mg/kg body weight) with a 20‐min interval between injections. The injection rate was 2 mL/s, which is lower than the rates (e.g., 5 mL/s) commonly used in human HP studies [3, 11, 14], to remain consistent with our ongoing patient studies that use a 2 mL/s injection rate to minimize the physiological burden and challenges associated with repeated intravenous access and bolus administration in vulnerable patients. The suitability of a 2 mL/s infusion rate for cardiac HP pyruvate studies is supported by prior vascular dynamic simulations and preliminary human investigations [19].

The ^1^H/^13^C‐integrated MR protocol included multislice 2D ^1^H cine with breath hold (#phase = 11–13, TE/TR = 1.712/4.442 ms, FOV = 400 × 400 mm^2^, matrix size = 512 × 512, slice thickness = 3 mm, #slice = 10), dynamic 2D ^13^C MRI with a spectral‐spatial RF pulse that selectively excite [1‐^13^C]pyruvate [18] (TE/TR = 18.42/588 ms, FOV = 400 × 400 mm^2^, matrix size = 40 × 40, slice thickness = 100 mm, #slice = 1, flip angle = 7.5°, #phase/cycle = 5), and dynamic ^13^C MRS (slice thickness = 100 mm, #slice = 1, flip angle = 7.5°, TR = 2 s, spectral width/points = 10 kHz/4096, #acquisition = 120). As a result, time‐resolved [1‐^13^C]pyruvate images were acquired using ^13^C MRI, while time‐resolved ^13^C spectra containing resonances from [1‐^13^C]pyruvate, [1‐^13^C]lactate, [1‐^13^C]alanine, and [^13^C]bicarbonate resonances were obtained using ^13^C MRS. Both ^1^H and ^13^C data were acquired along the short‐axis plane. ^13^C acquisitions were performed either without or with prospective ECG gating synchronized to the ES phase.

### 2.4 Ventricular Volume Measurement and Correlation With HP Pyruvate Signals

The endocardial borders of the RV and LV were contoured in the ^1^H cine images [20] using cvi42 (version 6.3.1; Circle Cardiovascular Imaging Inc., Calgary, Canada). The ventricular volume for each phase was calculated based on the number of voxels within the contour and the slice thickness. Cyclic change of the ventricular volumes, $V_{\mathrm{RV}}\left(\phi \right)$ and $V_{\mathrm{LV}}\left(\phi \right)$, were described as second‐order Fourier series [21] along cardiac phase, ϕ.

For time‐resolved HP [1‐^13^C]pyruvate MRI, correlation analysis was performed between HP [1‐^13^C]pyruvate intensity and ventricular volume using phase‐resolved pyruvate signals and the corresponding volumes across five cardiac cycles acquired during the stable decay period (35–50 s after injection; 80 acquisition points in total).

### 2.5 Hemodynamic Modeling of HP [1‐ ^13^C]Pyruvate

HP [1‐^13^C]pyruvate signal, S(t), was modeled as a sum of first‐pass component, $C_{1}\left(t\right)$, and a recirculation component, $C_{\mathrm{RC}}\left(t\right)$. The first‐pass component was described by a dual‐chamber model: $C_{1}\left(t\right)=C_{1,\mathrm{RV}}\left(t\right)+C_{1,\mathrm{LV}}\left(t\right)$. The LV component was assumed to be a scaled ($A_{\mathrm{RL}}$) and time‐shifted ($t_{\mathrm{RL}}$) analogue of the RV component (Equation 3). Similarly, the recirculation component was modeled as a sum of scaled ($A_{n}$) and time‐delayed ($t_{n}$) replica of the first‐pass components (Equation 4). In this study, we considered up to second passage (*N* = 2), considering the reliable observation time window.

(3) $$\begin{array}{c} C_{1,\mathrm{RV}}\left(t\right)=A_{\mathrm{RL}}\cdot C_{1,\mathrm{LV}}\left(t-t_{\mathrm{RL}}\right) \end{array}$$

(4) $$\begin{array}{c} C_{\mathrm{RC}}\left(t\right)=\sum_{n=2}^{N}A_{n}\cdot C_{1}\left(t-t_{n}\right) \end{array}$$

A multistage fitting algorithm was implemented to estimate the parameters. First, the hemodynamic model was fit to initial measurements (0–30 s) without the recirculation component. Second, scaling factor (i.e., $A_{2}$) and time delay (i.e., $t_{2}$) for recirculation were estimated from the residual signal, $S\left(t\right)-C_{1}\left(t\right)$. Finally, optimized parameters for both components were calculated from the full dataset using the Trust‐Region Reflective algorithm. All parameters were refined simultaneously to minimize the residual sum of squares between the model and the measured data. In vivo *T*_*1*_ was assumed consistent for each dataset.

### 2.6 Reconstruction of Phase‐Aligned HP Pyruvate Timecourse

A reconstruction algorithm was developed to estimate phase‐aligned pyruvate dynamics at a targeted cardiac phase, $\phi_{T}$. The algorithm demodulates the measured pyruvate timecourse, $S_{M}\left(t\right)$, and corrects the signal intensity to the estimated amplitude at $\phi_{T}$. To accomplish this, a time‐dependent modulation factor, M(t), is calculated as the ratio of the predicted signal intensity at the acquired cardiac phase to that at $\phi_{T}$ (Equation 5). The signal S(t) in the denominator is the ideal hemodynamic model described in the previous section. Finally, the phase‐aligned signal at $\phi_{T}$, $S_{R}\left(t\right)$, is calculated by Equation (6).

(5) $$\begin{array}{c} M\left(t,\phi \left(t\right)\right)=\frac{\sum_{n=1}^{\infty }\left(C_{n,\mathrm{RV}}\left(t\right)\cdot V_{\mathrm{RV}}\left(\phi \left(t\right)\right)+C_{n,\mathrm{LV}}\left(t\right)\cdot V_{\mathrm{LV}}\left(\phi \left(t\right)\right)\right)}{S\left(t\right)} \end{array}$$

(6) $$\begin{array}{c} S_{R}\left(t\right)=S_{M}\left(t\right)\cdot M\left(t,\phi_{T}\right) \end{array}$$

The theoretical upper and lower bounds of the HP pyruvate curves were estimated from phase‐aligned reconstructions at end‐diastole and end‐systole, respectively. Because the observed ^13^C MRS signals are contributed from both LV and RV pools and these phases may not be synchronized, participant‐specific ES and end‐diastolic (ED) phases were identified from an amplitude‐weighted biventricular volume curve ($W_{\mathrm{LV}}\cdot V_{\mathrm{LV}}\left(\phi \right)+W_{\mathrm{RV}}\cdot V_{\mathrm{RV}}\left(\phi \right)$), where the weights were derived from the kinetic model fitting. This global end‐diastole and end‐systole ensure that the reconstructed envelopes capture the full dynamic range of the total measured signal.

**FIGURE 1:**
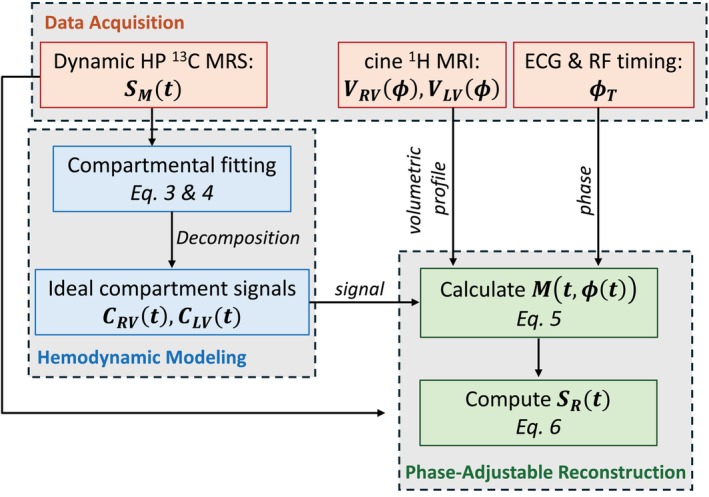
Stepwise workflow of the cardiac phase‐adjustable reconstruction pipeline. The framework integrates multimodal inputs (^1^H cine MRI, ECG timing, and ^13^C MRS) into a multicompartmental hemodynamic model. The theoretical kinetic components are subsequently utilized to compute a time‐dependent modulation factor (Equation 5), which retroactively aligns the measured non‐ECG‐triggered signals to a user‐defined target cardiac phase (Equation 6).

The stepwise workflow of the phase‐adjustable reconstruction pipeline is summarized in Figure 1. Hemodynamic modeling, phantom simulation, and human data reconstruction and analysis were performed using MATLAB (R2024b, The MathWorks, Natick, MA, USA).

## 3 Results

### 3.1 Hemodynamic Simulation of HP Pyruvate

**FIGURE 2:**
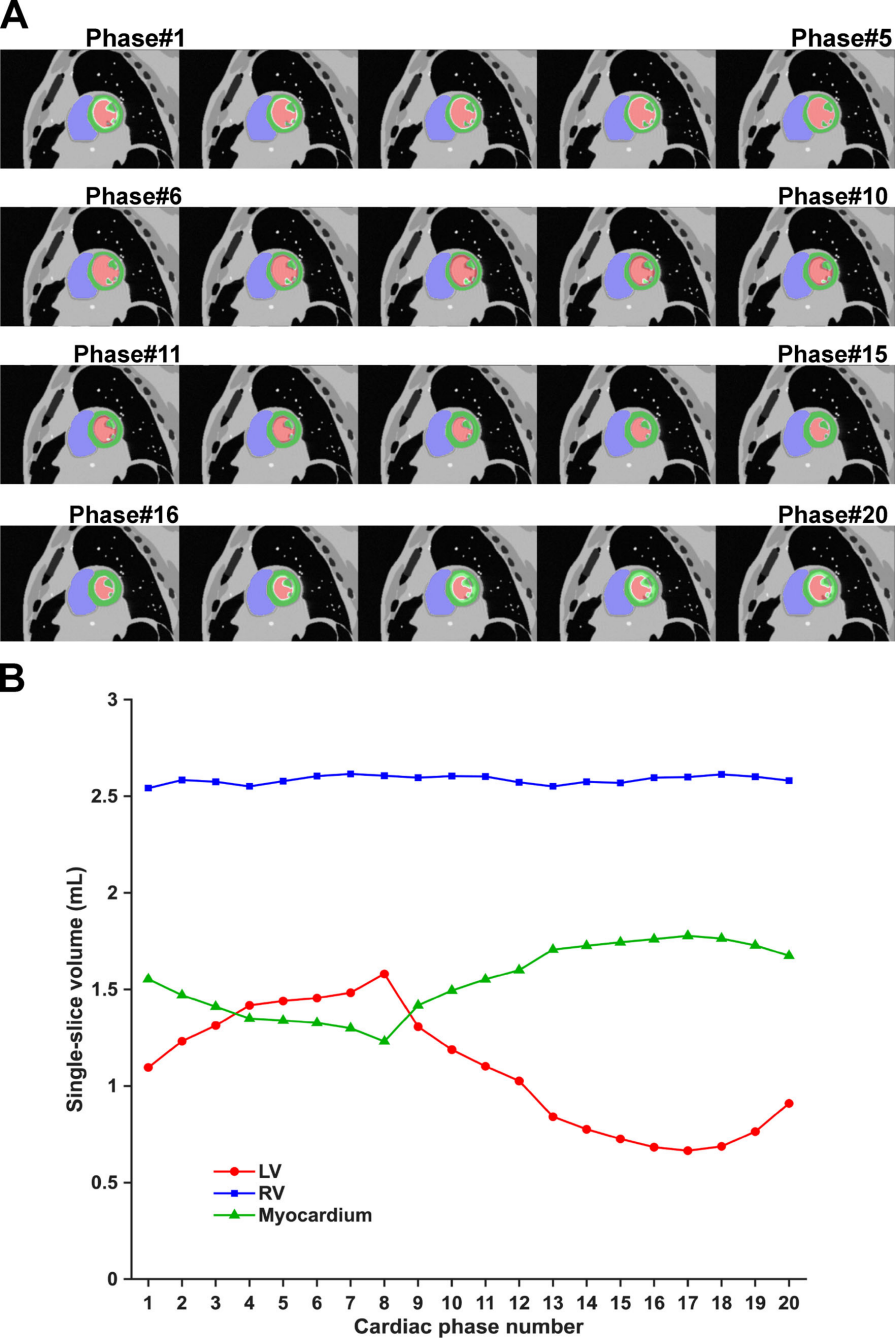
Cyclic volume changes of the digital cardiac phantom. (A) Simulated dynamic images of the MRXCAT phantom over a complete cardiac cycle (20 phases, default HR = 59 bpm). Segmentation masks are overlaid on the simulated anatomical images to delineate three compartments used for kinetic modeling: LV (red shaded region), RV (blue), and myocardium (green). (B) Compartmental volumes are plotted across the cardiac phases for the LV (red circles), RV (blue squares), and myocardium (green triangles). LV, left ventricle; RV, right ventricle.

**FIGURE 3:**
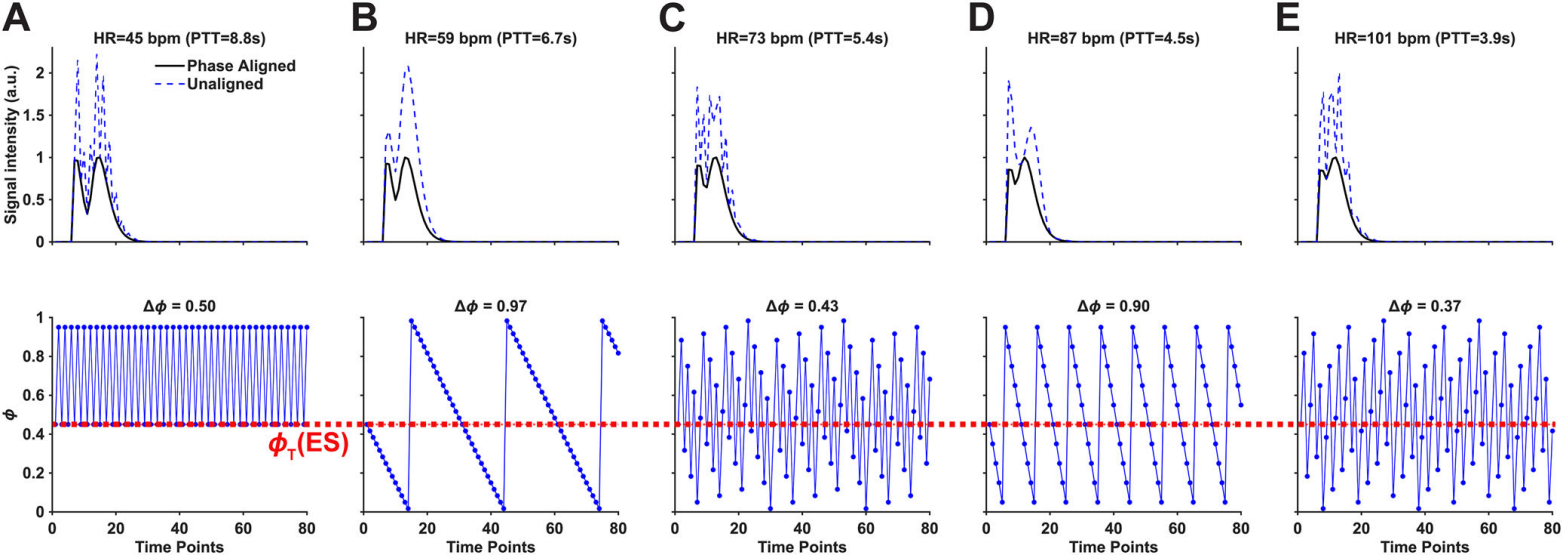
Simulation of cardiac phase misalignment and its impact on dynamic signal fidelity across a physiological range of heart rates. Simulation results are displayed for the heart rates of (A) 45, (B) 59, (C) 73, (D) 87, and (E) 101 bpm. A constant repetition time (TR = 2 s) was used for all HRs. (Top Row) Comparison of ideal, phase‐aligned dynamic curves (black solid lines) with unaligned signal trajectories (blue dashed lines). The unaligned curve shows signal oscillations caused by pseudo‐random cardiac‐phase sampling. The signal envelope was modulated using a biventricular total volume model derived directly from the digital phantom. (Bottom row) Corresponding temporal evolution of the cardiac sampling phase (ϕ), showing the drift of the sampled cardiac phase (Δϕ, blue connected markers) relative to the targeted end‐systole (red dotted line). Additional simulation results for heart rates of 38, 52, 66, 80, 94 bpm are available in Figure S1. ES, end‐systole; HR, heart rate; PTT, pulmonary transit time.

Time‐resolved anatomical images were generated from the digital cardiac phantom to depict volumetric profiles of LV, RV, and myocardium over the cardiac cycle, key cardiac compartments needed for HP pyruvate simulations, Figure 2. Numerical simulations with the phantom confirmed that pyruvate timecourse is significantly affected by HR and resulting misalignment from intended cardiac phase, Figure 3. The fixed 2‐s sampling period interacted with R‐R interval (RR), producing a beat‐frequency effect. The sampling pattern was governed by the cardiac phase increment, ∆ϕ, the fraction of the number of cardiac cycles elapsed during the sampling interval ($∆\phi =\mathrm{mod}\left(\frac{\mathrm{TR}}{\mathrm{RR}},1\right)$). A large ∆ϕ (e.g., near 0.5) induced a fast alternation in cardiac phase (e.g., systole vs. diastole). For instance, at HR of 45 bpm (RR = 1.33 s), the acquisition window skipped 1.5 cardiac cycles between consecutive TRs (∆ϕ = 2.0/1.33 = 1.5), creating an oscillating pattern. In contrast, HR of 73 bpm (RR = 0.82 s) and 101 bpm (RR = 0.59 s) resulted in the acquisition skipping approximately 2.43 (∆ϕ = 0.43) and 3.37 (∆ϕ = 0.37) cycles. These noninteger shifts produce complex, quasi‐random distributions of sampling points across the ventricular volume curve. The unaligned curves experienced severe amplitude distortion from the phase‐aligned reference (59 bpm). The overall shape of the dynamic curves shifted due to HR‐dependent changes in PTT, and the volume‐induced artifacts remained a dominant source of error across all simulated conditions. Additional simulation results at HRs of 38, 52, 66, 80, and 94 bpm are available in Figure S1.

### 3.2 Correlation Between HR and ECG Timing in Humans

**FIGURE 4:**
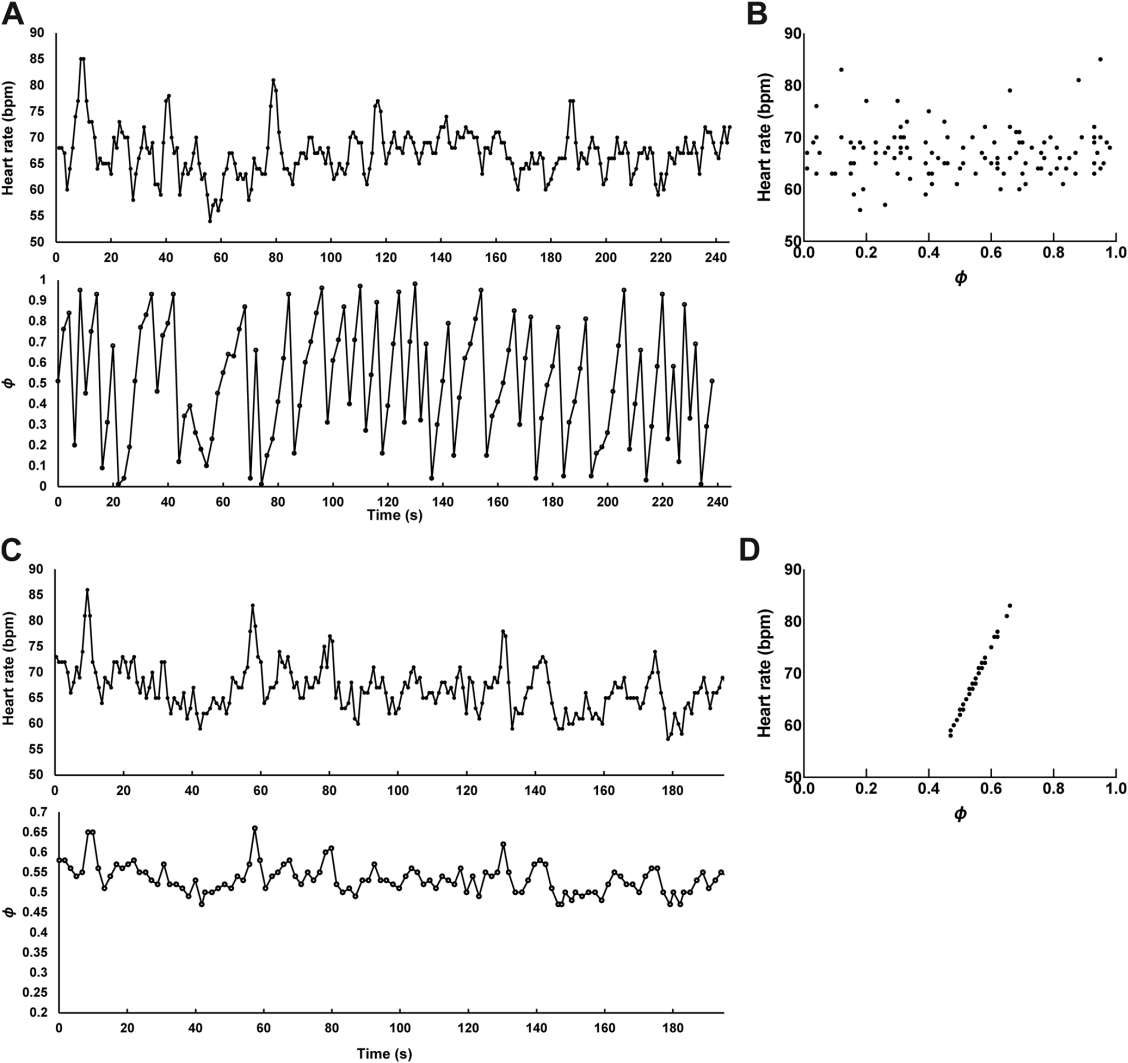
Impact of varying heart rate on cardiac phase in non‐ECG‐gated versus ECG‐gated acquisitions. Dynamic ^13^C MRS data were acquired from participant #2 following HP pyruvate injection without (A and B) and with (C and D) ECG gating. Temporal profiles of instantaneous HR (top row) and relative cardiac phase (ϕ) sampled at each RF excitation (bottom row) for injection (A) #1 and (C) #2. Scatter plots of instantaneous HR versus relative cardiac phase for injection (B) #1 and (D) #2. The coefficients of determination (*R* ^2^) are 0.002 for non‐ECG gated acquisition (injection 1) and 0.9950 for ECG‐gated acquisition (injection 2). Results from participant #1 are available in Figure S2. ϕ, cardiac phase; HP, hyperpolarized; HR, heart rate.

We evaluated the stability of synchronization between RF excitation and cardiac phase. Without ECG gating, the timing of RF excitation (and data collection) was asynchronous with the cardiac rhythm (coefficient of determination: *R* ^2^ = 0.003–0.004 for participant #1 and *R* ^2^ = 0.002 for participant #2), and the sampled cardiac phases were broadly distributed across the cardiac interval, Figure 4A,B. In contrast, ECG gating dramatically stabilized the cardiac phase at which data were acquired but showed strong coupling between HR and cardiac phase (*R* ^2^ = 0.9950), Figure 4C,D. Data from participant #1 are provided in Figure S2. These observations suggest that fixed trigger delay times in prospective gating may lead to phase variability in the presence of HR fluctuations.

### 3.3 Dynamic HP [1‐ ^13^C]Pyruvate Cardiac Imaging

**FIGURE 5:**
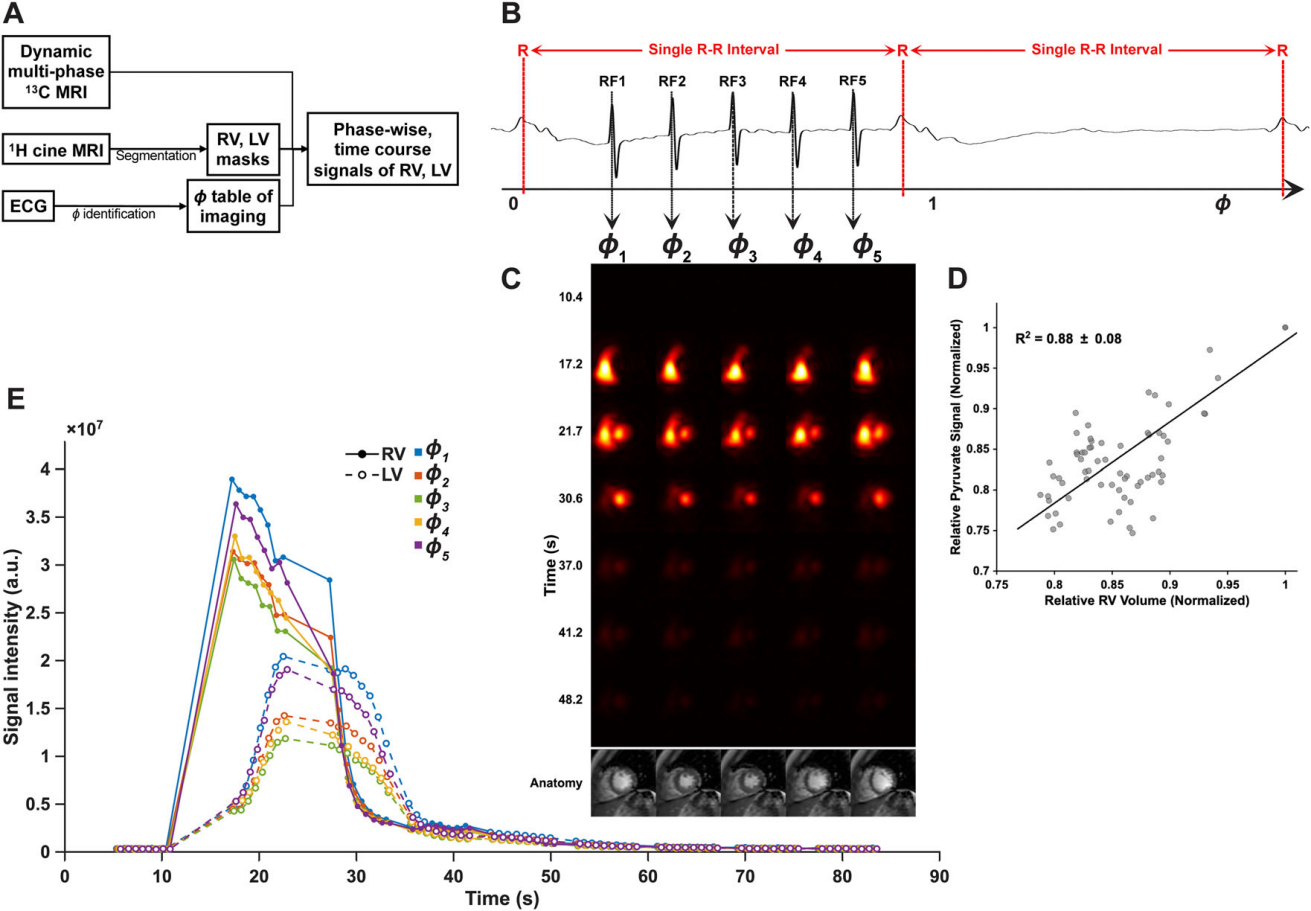
Dynamic HP [1‐^13^C]pyruvate cardiac imaging and dynamic ventricular timecourses. (A) Flowchart of the data processing pipeline. RV and LV masks derived from ^1^H cine MRI segmentation were applied to the dynamic multiphase ^13^C MRI data to extract phase‐wise timecourses utilizing an ECG‐derived ϕ table. (B) ECG trace detected sharp spikes (RF#1–5) that correspond to RF interference from the five RF excitations occurring within the heartbeat, indicating their associated cardiac phases. Red vertical lines denote a single R‐R interval. (C) Time‐resolved HP [1‐^13^C]pyruvate images from participant #1 (injection #3), displaying the spatiotemporal evolution of HP pyruvate bolus. (D) Scatter plot of the pyruvate signal across the cardiac phase identified from the ECG recording. (E) Reconstructed hemodynamic profiles of RV (solid lines) and LV (dashed lines) over a 90‐s duration at the acquired five cardiac phases. ϕ, cardiac phase; HP, hyperpolarized; LV, left ventricle; RV, right ventricle.

Dynamic, five‐phase ^13^C MRI of HP pyruvate was acquired from participant #1 with ECG‐gating by exciting pyruvate resonance using a spectral‐spatial RF pulse, Figure 5A. Sharp spikes appeared only when RF interference was picked up by the ECG leads during the five selective excitations, Figure 5B. Phase‐wise reconstruction illustrated a strong dependency of pyruvate signal intensity on cardiac phase, Figure 5C. A strong linear correlation was observed between measured signal intensity and ventricular volume (*R* ^*2*^ = 0.88 ± 0.08), particularly in the RV during the period of slow signal decay, supporting the assumption that ventricular volume can serve as a surrogate for the expected HP signal intensity within a given cardiac cycle, Figure 5D. Peak signal was consistently higher during diastole than systole in both ventricles, Figure 5E. Across all cardiac phases, the averaged signal peak time was 24.4 ± 0.4 s in the RV and 18.4 ± 0.1 s in the LV. The peak pyruvate signal intensity in the LV was 46.1% ± 6.3% of that in the RV.

**FIGURE 6:**
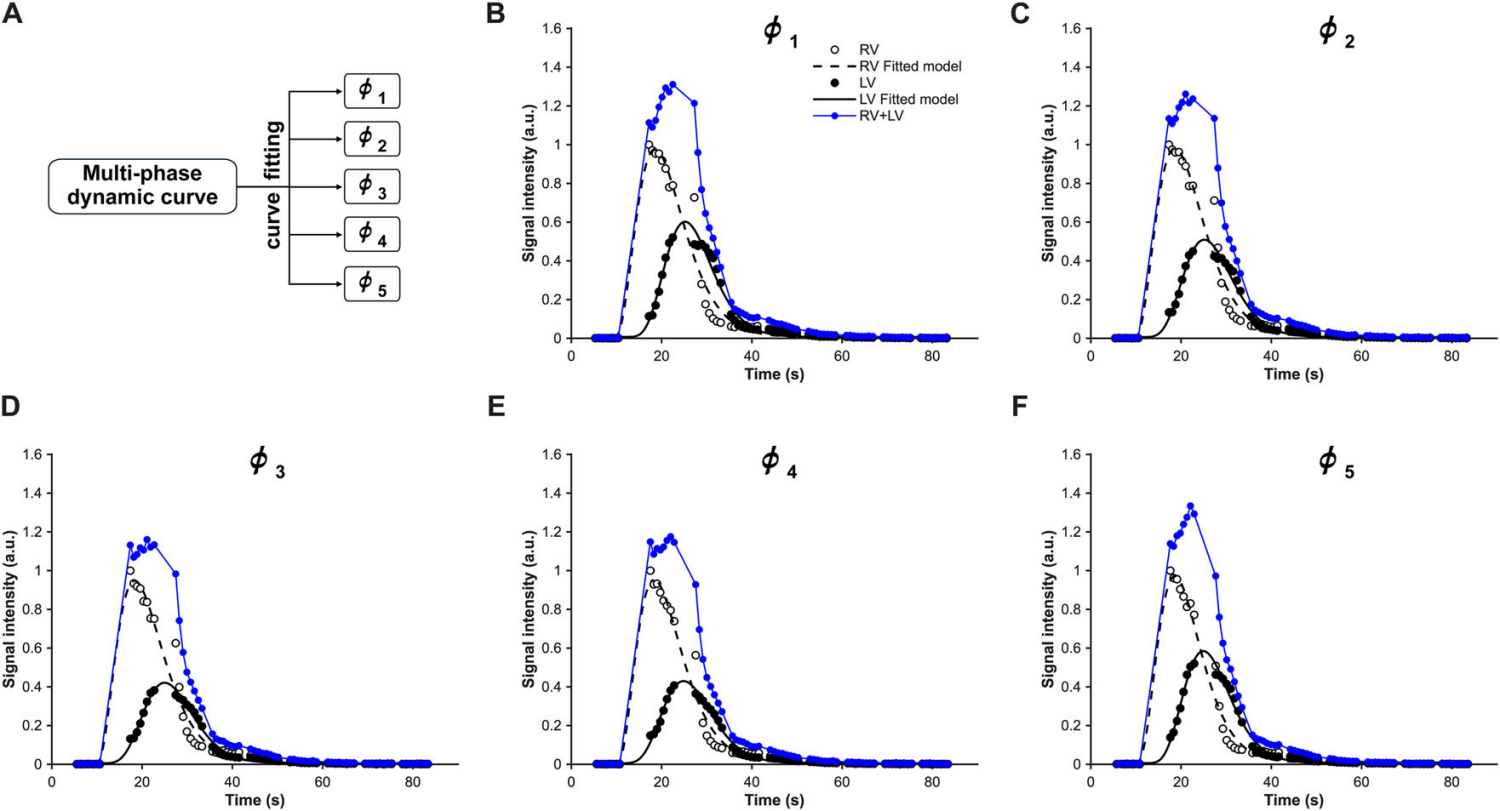
Phase‐wise kinetic model fitting of in vivo multiphase pyruvate data. (A) Dynamic pyruvate timecourses generated from multiphase ^13^C pyruvate MRI in Figure 4C were fitted to the hemodynamic model. (A) Schematic workflow illustrating the independent curve fitting performed for each of the five acquired cardiac phases (ϕ = 0.25, 0.38, 0.50, 0.65, and 0.80). (B–F) Modeling results corresponding to five cardiac phase: (B) 0.25, (C) 0.38, (D) 0.50, (E) 0.65, and (F) 0.80. Measured signal intensities for RV (hollow circles) and LV (solid circles) are plotted together with their fitted gamma‐variate models (RV: Dashed black line; LV: Solid black line). The RV‐LV combined measurements are displayed in blue.

The image‐derived pyruvate timecourses could be described using the proposed hemodynamic model with excellent fit (*R* ^2^ = 0.9800 ± 0.0062 for RV, 0.9864 ± 0.0043 for LV), Figure 6. The image‐based PTT was estimated to be 6.5 ± 0.2 s at an average HR of 75.7 ± 3.8 bpm. Detailed fitting parameters are summarized in Table S1.

### 3.4 Compartmental Decomposition of HP Pyruvate From in Vivo ^13^C MRS

The proposed signal analysis pipeline was applied to four in vivo dynamic HP [1‐^13^C]pyruvate MRS datasets, comprising three non‐ECG‐gated and one prospective ECG‐gated acquisition. Application of the multistage gamma‐variate model revealed that a three‐component structure comprising the RV first‐pass, LV first‐pass, and a global recirculation component was essential for an accurate description of the measured signal. The model consistently achieved an excellent fit across all datasets, with coefficients of determination (*R* ^2^) ranging from 0.9783 to 0.9949 (Table 2), demonstrating its robustness against physiological variations and cardiac motion artifacts.

**TABLE 2:** Hemodynamic parameters for dynamic HP ^13^C MRS.

Participant ID	1		2	
Injection#	1	2	1	2
Acquisition type	Nongated	Nongated	Nongated	ECG‐gated
HR (bpm)	72.4	74.8	67.4	67.5
PTT (s)	6.79	6.79	8.69	7.06
LV/RV volume ratio (%)	47.2	48.4	47.8	48.7
Recirculation/first passage amplitude ratio (%)	16.7	15.7	19	15.5
*R* ^2^ (goodness of fit)	0.9859	0.9783	0.9917	0.9949

**FIGURE 7:**
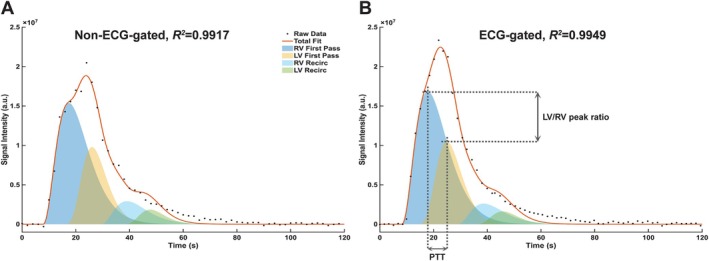
Compartmental decomposition of HP [1‐^13^C]pyruvate from time‐resolved MRS acquisitions in participant #2. (A) Nongated (injection #1) and (B) ECG‐gated (injection #2) ^13^C MRS data (black dots) acquired following HP pyruvate were fitted using a multicompartmental kinetic model (solid red line). Blue and yellow shaded regions represent first‐pass RV and LV signals, respectively. Light blue and green shaded regions represent systemic recirculation. Panel B includes annotations for hemodynamic metrics, including peak times (vertical dashed lines), PTT (horizontal arrow), and the LV/RV peak signal ratio (vertical arrow). Fitting results for participant #1 are available in Figure S3. HP, hyperpolarized; LV, left ventricular; PTT, pulmonary transit time; RV, right ventricular.

Figure 7 shows HP pyruvate timecourses of participant #2 decomposed to first‐pass and second‐pass RV and LV components. The nongated timecourse (Figure 7A) had stronger signal oscillations than the ECG‐gated timecourse, which was synchronized to the ES phase (Figure 7B). Despite these fluctuations, the kinetic model effectively extracted the underlying bolus envelope, outlining the temporal delay and dispersion of the LV bolus compared to the RV. The model fit (*R* ^2^ = 0.9917 for nongated and 0.9949 for gated data) confirmed the distinct physiological separation of the ventricular compartments, free from the confounding influence of large‐volume changes. Compartmental decomposition and fitting results for participant #1 are shown in Figure S3.

Quantitative analysis of the fitted parameters revealed consistent hemodynamic metrics. PTT derived from the delay between the RV and LV peaks was 6.79 s for participant #1 (consistent across two injections) and ranged from 7.06 to 8.69 s for participant #2. The LV signal amplitude was 48.0% ± 0.7%, reflecting the expected dilution and *T*_*1*_ relaxation during pulmonary transit. The recirculation component accounted for approximately 15%–19% of the total signal integral, explaining the persistent signal tail at later time points. Hemodynamic parameters are summarized in Table 2.

### 3.5 Phase‐Adjustable Reconstruction of Pyruvate Hemodynamics

**FIGURE 8:**
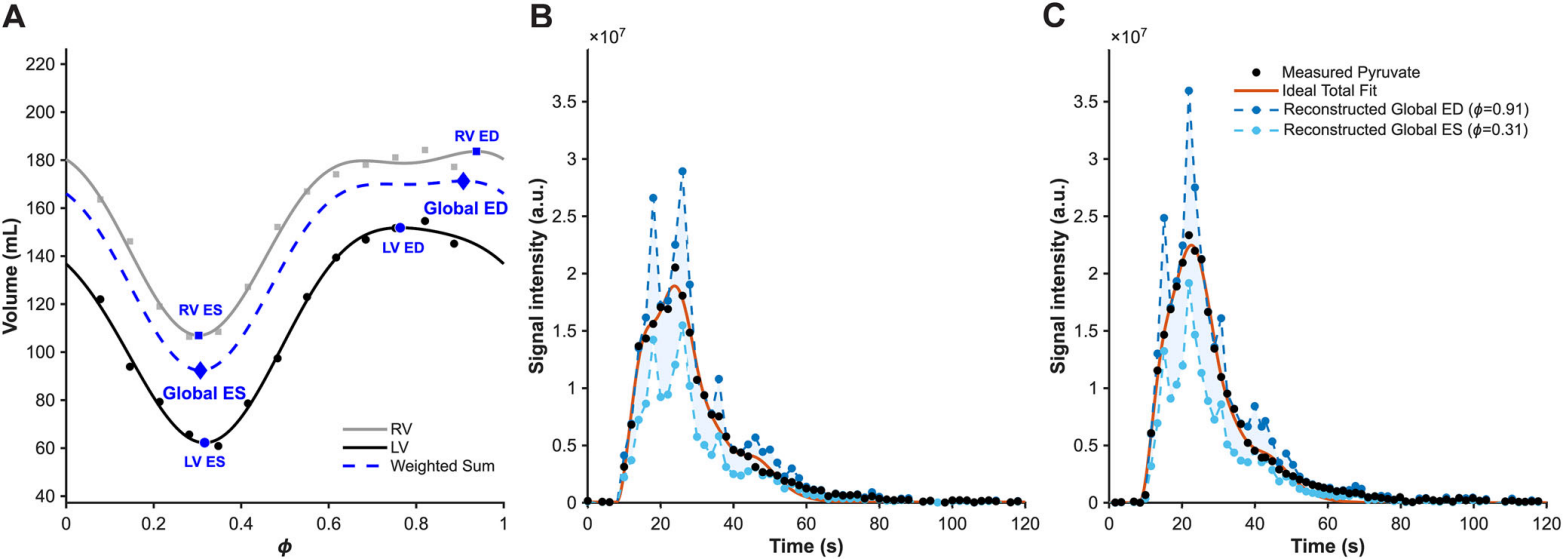
Characterization of cardiac‐induced signal modulation and reconstruction in participant #2. (A) Ventricular volume profile models derived from structural cine imaging. Temporal variations in RV (gray line) and LV (black line) volumes are shown over a single cardiac cycle, alongside the original discrete contouring data points. An amplitude‐weighted biventricular volume curve (blue dashed line) was calculated to determine the global ED and global ES phases (blue diamonds). (B and C) Visualization of the cardiac‐induced signal envelope for nongated (B) and ECG‐gated acquisition (C). Dashed dark‐blue and dashed light‐blue lines represent the reconstructed signal trajectories at global ED (ϕ = 0.91) and global ES (ϕ = 0.31), respectively. The shaded region indicates the dynamic range of signal modulation due to cardiac stroke volume. The solid red line indicates the ideal kinetic fit, and black markers denote the measured pyruvate signal. Reconstructed data from participant #1 are available in Figure S4. ED, end‐diastole; ES, end‐systole; LV, left ventricular; RV, right ventricular.

To validate the physiological basis of the proposed signal correction algorithm, subject‐specific cyclic profiles of cardiac volume were derived from ^1^H cine. As shown in Figure 8A, these normalized ventricular volume profiles characterize temporal variation in chamber volume across the cardiac cycle and serve as physiological templates for calculating phase‐dependent modulation factors. Using these volume profiles, both ECG‐gated and nongated datasets were inspected to validate the correction strategy. To visualize the full hemodynamic envelope of the HP signal, trajectories corresponding to ED (maximum volume) and ES (minimum volume) phases were reconstructed. The reconstructed timecourses define the upper and lower boundaries of signal intensity, with the shaded area quantifying instantaneous signal fluctuations driven by stroke volume, Figure 8B,C. In the ECG‐gated acquisition, signal was measured at the end‐systole phase (black markers). The measured data aligned closely with the theoretical ideal timecourse (red line) and remained consistent with the specific phase trajectory targeted during acquisition. Phase‐resolved reconstruction for participant #1 is included in Figure S4.

## 4 Discussion

In this study, we developed a comprehensive hemodynamic model to describe the in vivo HP [1‐^13^C]pyruvate timecourse in the heart. The model accounts for shifts in cardiac phase arising from varying HR or inaccuracies in trigger delay estimation. By integrating ECG timing recorded during ^13^C acquisition with independently acquired cardiac ^1^H cine, this model enables retrospective correction of cardiac phase misalignments and reconstruction of HP timecourses at a user‐defined cardiac phase of interest.

Our results demonstrate that ventricular volume serves as a reliable surrogate for HP pyruvate signal intensity within the cardiac blood pool. The linear correlation between measured cardiac pyruvate signal and ventricular volume suggests that the observed pyruvate signal is mainly governed by blood volume rather than intracavitary flow dynamics. This correlation allows for subject‐specific cine MRI to serve as a robust, personalized physiological template for calculating phase‐dependent modulation factors.

Hemodynamic analysis and cardiac phase alignment are crucial for the quantitative assessment of HP signals. In this study, HR was found to be sensitive to pyruvate bolus injection, particularly during the first ˜20 s of acquisition, coinciding with bolus arrival. This observation suggests that prospective ECG‐gating with a precalculated trigger delay may be insufficient to maintain phase coherence throughout the ^13^C acquisition. Therefore, the proposed method is not only essential for nongated acquisition but also serves as a critical refinement tool for correcting residual phase‐related artifacts in standard clinical ECG‐gated protocols.

The in vivo feasibility of the proposed hemodynamic model is supported by the successful signal phase‐adjustment and multicompartmental decomposition, as demonstrated in Figure 6 and the gated reconstructions in Figure 8. The multiphase ^13^C pyruvate MRI‐derived LV‐to‐RV signal ratio closely matched the model‐based ratio, reflecting signal dilution and *T*_*1*_ relaxation during pulmonary transit. Inclusion of recirculation components strengthened the model by fitting the late‐time signal tail compared with simpler dual‐gamma variants, consistent with gadolinium‐based cardiac studies [15]. The PTT derived from the model fitting also agreed with independent image‐based measurements, confirming that the model effectively disentangles the composite cardiac signal into its distinct physiological components. The PTT was higher than values reported from a prior gadolinium perfusion study (4.83–5.11 s) [17]. This discrepancy may reflect physiological differences between study participants, but may also arise from differences in modeling strategies. For instance, exclusion of the recirculation component, as often done in prior studies, can artificially broaden the apparent first‐pass profile and bias PTT estimation.

A powerful implication of this work is its utility to assess cardiac metabolism using HP ^13^C pyruvate without being confounded by HR variability. The retrospective, volume‐guided correction approach reduces reliance on prospective ECG triggering, a common source of variability. This approach may be particularly valuable in patients with arrhythmias or other conditions associated with HR instability, where conventional gating strategies are unreliable. Moreover, because the proposed reconstruction compensates for cardiac phase effects and enables multiphase analysis, it will become essential for unifying HP cardiac datasets acquired using different MR protocols or across multiple sites. Since cardiac phase misalignment can introduce substantial physiological variability that may obscure subtle metabolic differences associated with disease or therapeutic interventions, the proposed correction is expected to reduce this variability, enhancing sensitivity to disease‐related metabolic alterations and improving statistical power while potentially reducing required sample sizes.

We acknowledge several limitations in this study and directions for future work. *First*, the cohort size remains small, and larger studies are required to establish statistical robustness and generalizability. Extension of this approach to patient cohorts with cardiac disease will further validate the method, as HR variability and ventricular volume changes are more pronounced under pathological conditions. *Second*, this study focused on HP pyruvate and did not model metabolic conversion to downstream products. Because these metabolic products predominantly arise from the myocardium and contribute relatively small fractions compared with the blood pool (Table S2), we anticipate minimal impact on the hemodynamic model. Nevertheless, incorporating HP alanine, lactate, and bicarbonate would further strengthen the framework and enable comprehensive characterization of phase‐dependent HP timecourses in the heart. *Third*, while our empirical data (Figure 5D) demonstrate a linear relationship between signal and bulk ventricular volume during the stable signal‐decay period, this relationship may deviate from strict linearity during the initial, highly turbulent bolus arrival, or in pathological states with severe intracavitary flow anomalies. *Fourth*, our current framework relies on ^1^H cine MRI to derive the cyclic profile of ventricular volumes. Although ^1^H cine MRI is routinely included in cardiac MR protocols, simultaneous ^1^H and ^13^C acquisition or self‐navigating ^13^C acquisition would reduce the total acquisition time and improve robustness by enabling real‐time volumetric tracking synchronized with the metabolic data.

Additionally, our current study did not account for respiratory motion or other physiologic variations. For the human study, respiratory artifacts were minimized by instructing subjects to maintain shallow breathing and by using a thick slab for the ^13^C acquisitions to ensure that the heart remains within the excitation volume. However, for future translation to high‐resolution thin‐slice imaging, or in patients unable to maintain stable shallow breathing, respiratory‐induced motion could compromise the robustness of the volume‐guided correction. Future developments incorporating respiratory navigation will be necessary to decouple cardiac and respiratory modulations in those advanced acquisitions. *Finally*, despite the excellent stability and goodness‐of‐fit of the global multicompartmental fitting approach in healthy cohorts, its performance can depend on the SNR of the HP [1‐^13^C]pyruvate bolus. In clinical scenarios with compromised bolus delivery or low SNR, separating the lower‐amplitude recirculation tail from the first‐pass kinetics may become more susceptible to noise‐induced fitting instabilities. Future simulations that incorporate recirculation under reduced‐SNR conditions will further evaluate the robustness of the multicompartmental fitting framework.

## 5 Conclusion

This study establishes a hemodynamic model of HP pyruvate in the human heart. By integrating this model with ECG recordings and cardiac ^1^H cine imaging, we developed a novel, volume‐guided retrospective cardiac phase correction algorithm to mitigate artifacts arising from cardiac phase misalignment. Together, these advances provide a foundation for more accurate quantification of HP cardiac metabolic imaging. To our knowledge, this work presents one of the first hemodynamic models specifically developed for and validated in the complex hemodynamic environment, addressing a key bottleneck in quantitative cardiac HP analysis. Ultimately, this work enables more robust metabolic imaging of the heart and is applicable to data acquired with or without prospective cardiac triggering. Although not demonstrated here due to limited signal sensitivities, the proposed framework is extendable to downstream in vivo products such as HP [1‐^13^C]lactate, [1‐^13^C]alanine, and [^13^C]bicarbonate. Moreover, the retrospective phase‐adjustable reconstruction scheme can be further extended to *imaging* datasets.

## Funding

This work was supported by the National Institute of Biomedical Imaging and Bioengineering (P41EB015908), the NIH Office of the Director (S10OD018468), the National Heart, Lung, and Blood Institute (R01HL170039), the National Center for Research Resources (S10RR029119), and the Muscular Dystrophy Association (MDA963281).

## Supporting information


**Figure S1:** Extended simulation of heart rate variability effects on dynamic signal acquisition. Simulation results are displayed for the five heart rates not shown in Figure 2: (A) 38, (B) 52, (C) 66, (D) 80, and (E) 94 bpm. A constant TR of 2 s was used. (Top row) Comparison of ideal, phase‐aligned dynamic curves (black solid lines) with unaligned signal trajectories (blue dashed lines). The unaligned curve shows signal oscillations caused by pseudo‐random cardiac‐phase sampling. The signal envelope was modulated using a biventricular total volume model derived directly from the digital phantom. (Bottom row) Corresponding temporal evolution of the cardiac sampling phase (ϕ), showing the drift of the sampled cardiac phase (Δϕ, blue connected markers) relative to the targeted end‐systole (red dotted line). ES, end‐systole; HR, heart rate; PTT, pulmonary transit time.
**Figure S2:** Cardiac phase sampling dynamics for participant #1. Two non‐ECG‐gated MRS acquisitions were performed with HP pyruvate injections. (Top row) Temporal profiles of instantaneous HR and (bottom row) relative cardiac phase (ϕ) sampled at each RF excitation for injection (A) #1 and (C) #2. Scatter plots of instantaneous HR versus relative cardiac phase for injection (B) #1 and (D) #2. The coefficients of determination (*R* ^2^) are 0.004 for injection 1 and 0.003 for injection 2. Both datasets demonstrate negligible correlation between HR and sampled phase, confirming the pseudo‐random sampling characteristic of the nongated free‐running acquisition. HP, hyperpolarized; HR, heart rate.
**Figure S3:** Compartmental decomposition of HP [1‐^13^C]pyruvate from non‐ECG‐gated MRS acquisitions in participant #1. Time‐resolved, non‐ECG‐gated ^13^C MRS data (black dots) acquired following HP pyruvate were fitted using a multicompartmental kinetic model (solid red line). (A) Injection #1 and (B) injection #2. Blue and yellow shaded regions represent first‐pass RV and LV signals, respectively. Light blue and green shaded regions represent systemic recirculation. HP, hyperpolarized; LV, left ventricular; RV, right ventricular.
**Figure S4:** Characterization of cardiac‐induced signal modulation and reconstruction in participant #1. (A) Ventricular volume profile models derived from structural cine imaging. Temporal variations in RV (gray line) and LV (black line) volumes are shown over a single cardiac cycle, alongside the original discrete contouring data points. An amplitude‐weighted biventricular volume curve (blue dashed line) was calculated to determine the global ED and global ES phases (blue diamonds). (B and C) Visualization of the cardiac‐induced signal envelope for injection 1 (B) and 2 (C). Dashed dark‐blue and dashed light‐blue lines represent reconstructed signal trajectories at global ED and global ES, respectively. The shaded region indicates the dynamic range of signal modulation due to cardiac stroke volume. The solid red line indicates the ideal kinetic fit, and black markers denote the measured pyruvate signal. ED, end‐diastole; ES, end‐systole; LV, left ventricular; RV, right ventricular.
**Table S1:**. Hemodynamic model parameters used in Figure 5 from participant #1.
**Table S2:** Normalized HP ^13^C products. Metabolic products were quantified from time‐averaged ^13^C spectra, and normalized to the total HP ^13^C signals (TC), the sum of [1‐^13^C]pyruvate, [1‐^13^C]pyruvate‐hydrate, [1‐^13^C]lactate, [1‐^13^C]alanine, and [^13^C]bicarbonate.

## Data Availability

The MATLAB code for the hemodynamic modeling, fitting algorithms, and reconstruction framework is available at: https://github.com/Sung‐hanLin/Hyperpolarized‐Cardiac‐Reconstruction.
