# Supplementary material for: Hemodynamic Modeling and Phase‐Adjustable Reconstruction of Hyperpolarized Cardiac 13C MRS Using 1H Cine and ECG Timing
Source: Magn Reson Med. 2026 May 10;96(3):1054–65. doi: 10.1002/mrm.70426 (PMC13327463; doi:10.1002/mrm.70426)
Supplement: Supplementary file 1 — Figure S1: Extended simulation of heart rate variability effects on dynamic signal acquisition. Simulation results are displayed for the five heart rates not shown in Figure 2: (A) 38, (B) 52, (C) 66, (D) 80, and (E) 94 bpm. A constant TR of 2 s was used. (Top row) Comparison of ideal, phase‐aligned dynamic curves (black solid lines) with unaligned signal trajectories (blue dashed lines). The unaligned curve shows signal oscillations caused by pseudo‐random cardiac‐phase sampling. The signal envelope was modulated using a biventricular total volume model derived directly from the digital phantom. (Bottom row) Corresponding temporal evolution of the cardiac sampling phase (ϕ), showing the drift of the sampled cardiac phase (Δϕ, blue connected markers) relative to the targeted end‐systole (red dotted line). ES, end‐systole; HR, heart rate; PTT, pulmonary transit time. Figure S2: Cardiac phase sampling dynamics for participant #1. Two non‐ECG‐gated MRS acquisitions were performed with HP pyruvate injections. (Top row) Temporal profiles of instantaneous HR and (bottom row) relative cardiac phase (ϕ) sampled at each RF excitation for injection (A) #1 and (C) #2. Scatter plots of instantaneous HR versus relative cardiac phase for injection (B) #1 and (D) #2. The coefficients of determination (R 2) are 0.004 for injection 1 and 0.003 for injection 2. Both datasets demonstrate negligible correlation between HR and sampled phase, confirming the pseudo‐random sampling characteristic of the nongated free‐running acquisition. HP, hyperpolarized; HR, heart rate. Figure S3: Compartmental decomposition of HP [1‐13C]pyruvate from non‐ECG‐gated MRS acquisitions in participant #1. Time‐resolved, non‐ECG‐gated 13C MRS data (black dots) acquired following HP pyruvate were fitted using a multicompartmental kinetic model (solid red line). (A) Injection #1 and (B) injection #2. Blue and yellow shaded regions represent first‐pass RV and LV signals, respectively. Light blue and green shaded [file MRM-96-1054-s001.pdf]

Supporting Information

Title: Hemodynamic modeling and phase-adjustable reconstruction of hyperpolarized cardiac ^{13}C MRS using ^{1}H cine and ECG timing

Authors: Sung-Han Lin[1], Corey Mozingo[1], Crystal E. Harrison[1], Kelley A. Derner[1], Craig R. Malloy[1,2,3], Jae Mo Park[1,3,4,5*]

Affiliations:

1. Advanced Imaging Research Center, UT Southwestern Medical Center, Dallas, Texas, USA 75390
2. VA North Texas Healthcare System, Dallas, Texas, USA 75216
3. Department of Radiology, UT Southwestern Medical Center, Dallas, Texas, USA 75390
4. Department of Biomedical Engineering, UT Southwestern Medical Center, Dallas, Texas, USA 75390
5. Charles and Jane Pak Center for Mineral Metabolism and Clinical Research, UT Southwestern Medical Center, Dallas, Texas, USA 75390

Correspondence to:

Jae Mo Park, Ph.D.

5323 Harry Hines Blvd. Dallas Texas, USA 75390

jaemo.park@utsouthwestern.edu

+1-214-645-7206

Supporting Figures

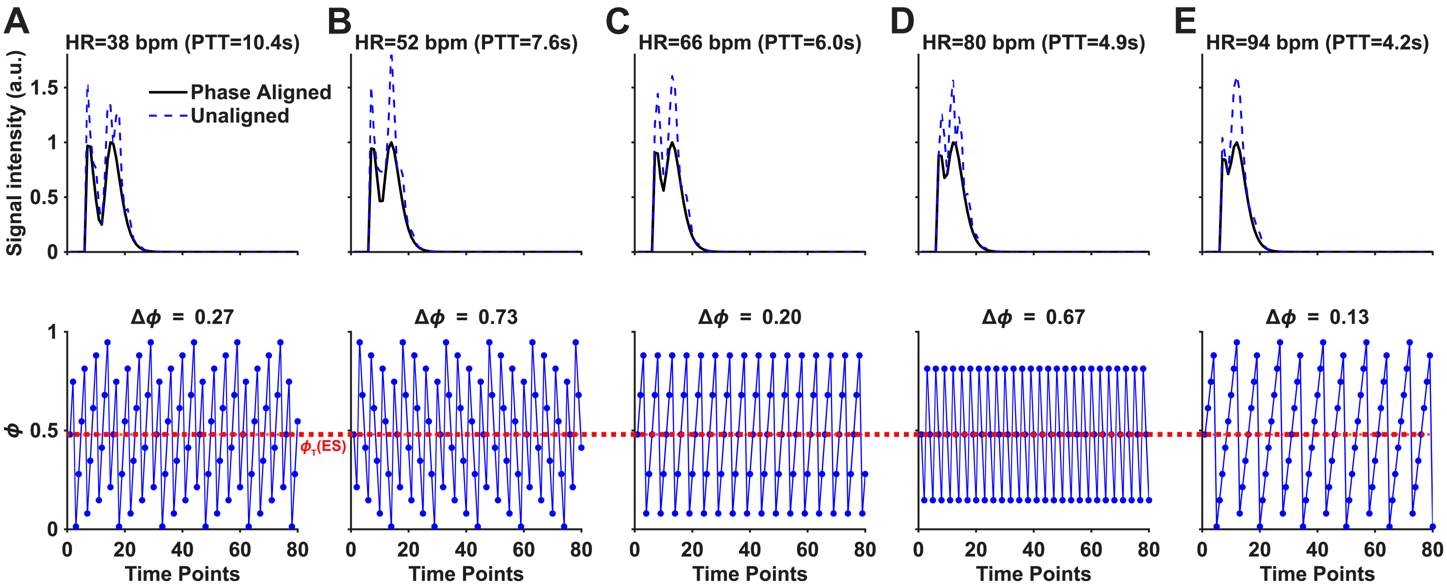

Figure S1. Extended simulation of heart rate variability effects on dynamic signal acquisition. Simulation results are displayed for the five heart rates not shown in Figure 2: (A) 38, (B) 52, (C) 66, (D) 80, and (E) 94 bpm. A constant TR of 2 s was used. (Top row) Comparison of ideal, phase-aligned dynamic curves (black solid lines) with unaligned signal trajectories (blue dashed lines). The unaligned curve shows signal oscillations caused by pseudo-random cardiac-phase sampling. The signal envelope was modulated using a biventricular total volume model derived directly from the digital phantom. (Bottom row) Corresponding temporal evolution of the cardiac sampling phase (ϕ), showing the drift of the sampled cardiac phase ($\Delta\phi$, blue connected markers) relative to the targeted end-systole (red dotted line). ES, end-systole; HR, heart rate; PTT, pulmonary transit time.

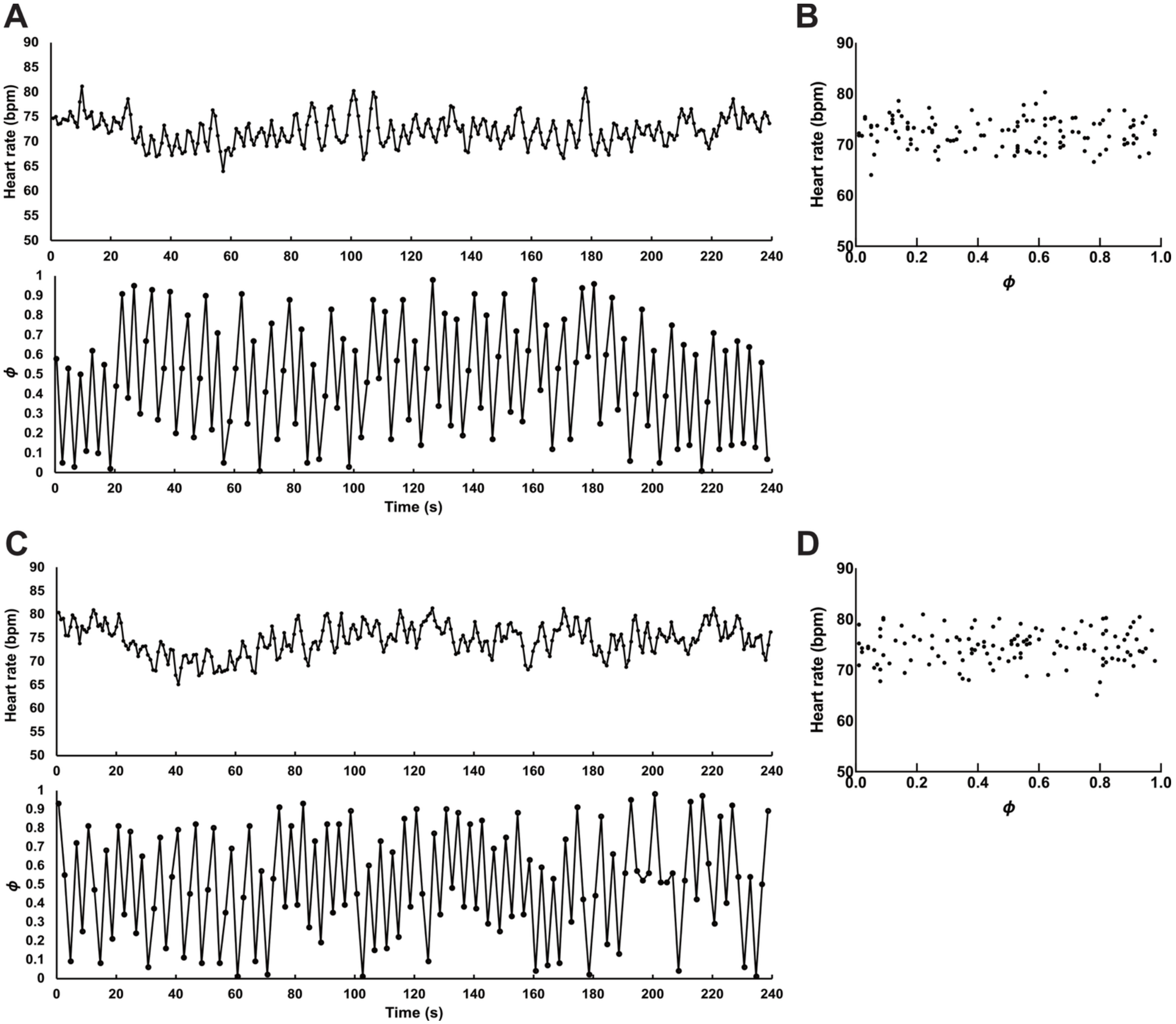

Figure S2. Cardiac phase sampling dynamics for participant #1. Two non-ECG-gated MRS acquisitions were performed with HP pyruvate injections. (Top row) Temporal profiles of instantaneous HR and (bottom row) relative cardiac phase (ϕ) sampled at each RF excitation for injection (A) #1 and (C) #2. Scatter plots of instantaneous HR versus relative cardiac phase for injection (B) #1 and (D) #2. The coefficients of determination (R^2) are 0.004 for injection 1 and 0.003 for injection 2. Both datasets demonstrate negligible correlation between HR and sampled phase, confirming the pseudo-random sampling characteristic of the non-gated free-running acquisition. HP, hyperpolarized; HR, heart rate.

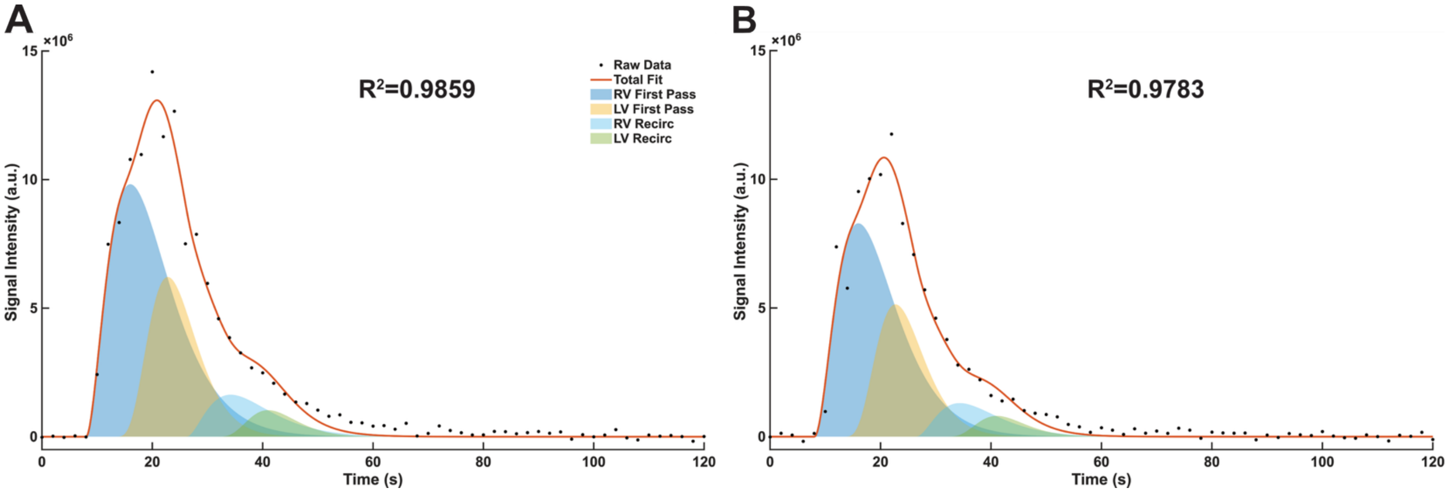

Figure S3. Compartmental decomposition of HP [1-^{13}C]pyruvate from non-ECG-gated MRS acquisitions in participant #1. Time-resolved, non-ECG-gated ^{13}C MRS data (black dots) acquired following HP pyruvate were fitted using a multi-compartmental kinetic model (solid red line). (A) Injection #1 and (B) injection #2. Blue and yellow shaded regions represent first-pass RV and LV signals, respectively. Light blue and green shaded regions represent systemic recirculation. HP, hyperpolarized; LV, left ventricular; RV, right ventricular.

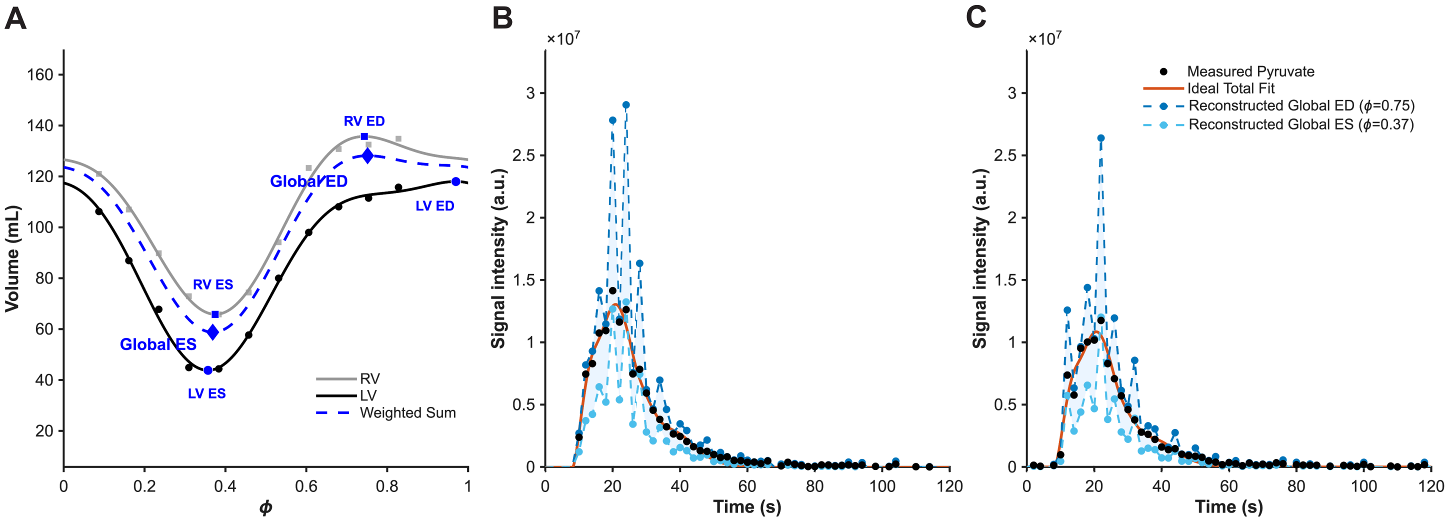

Figure S4. Characterization of cardiac-induced signal modulation and reconstruction in participant #1. (A) Ventricular volume profile models derived from structural cine imaging. Temporal variations in RV (gray line) and LV (black line) volumes are shown over a single cardiac cycle, alongside the original discrete contouring data points. An amplitude-weighted biventricular volume curve (blue dashed line) was calculated to determine the global ED and global ES phases (blue diamonds). (B, C) Visualization of the cardiac-induced signal envelope for injection 1 (B) and 2 (C). Dashed dark-blue and dashed light-blue lines represent reconstructed signal trajectories at global ED and global ES, respectively. The shaded region indicates the dynamic range of signal modulation due to cardiac stroke volume. The solid red line indicates the ideal kinetic fit, and black markers denote the measured pyruvate signal. ED, end-diastole; ES, end-systole; LV, left ventricular; RV, right ventricular.

SUPPORTING TABLES

Table S1. Hemodynamic model parameters used in Figure 5 from participant #1.

**Cardiac Phase (ϕ)**	**0.25**		**0.38**		**0.5**		**0.65**		**0.8**	
**Ventricles**	**R**	**L**	**R**	**L**	**R**	**L**	**R**	**L**	**R**	**L**
k	4.12	8.74	3.96	9.30	3.73	9.53	3.76	9.45	4.39	8.52
θ	3.30	2.00	3.42	2.00	3.50	2.00	3.35	2.00	2.86	2.00
**Arrival time (s)**	9.31	10.71	9.58	9.62	9.82	9.03	10.07	9.06	9.82	10.87
**R^2 (goodness of fit)**	0.972	0.980	0.976	0.984	0.980	0.989	0.985	0.990	0.987	0.989
**PTT (s)**	6.71		6.39		6.71		6.40		6.24	
**Peak intensity (RV/LV)**	1.62		1.93		2.25		2.22		1.66	

*PTT: pulmonary transit time, RV: right ventricle, LV: left ventricle

Table S2. Normalized HP ^{13}C products. Metabolic products were quantified from time-averaged ^{13}C spectra, and normalized to the total HP ^{13}C signals (TC), the sum of [1-^{13}C]pyruvate, [1-^{13}C]pyruvate-hydrate, [1-^{13}C]lactate, [1-^{13}C]alanine, and [^{13}C]bicarbonate.

Participant ID	Injection#	[^{13}C]Bicarbonate/TC	[1-^{13}C]Lactate/TC	[1-^{13}C]Alanine/TC
1	1	0.0204	0.0686	0.0393
1	2	0.0166	0.0674	0.0370
2	1	0.0142	0.0610	0.0385
2	2	0.0139	0.0588	0.0307
	Mean	1.63%	6.39%	3.64%
	Standard Deviation	0.30%	0.48%	0.39%
